# CD45RO-Positive Memory T-Cell Density in the Tumoral Core and Invasive Margin Predict Long-Term Survival in Esophageal Squamous Cell Carcinoma

**DOI:** 10.1245/s10434-024-16530-z

**Published:** 2024-12-05

**Authors:** Toshiki Noma, Tomoki Makino, Kenji Ohshima, Kotaro Yamashita, Takuro Saito, Koji Tanaka, Kazuyoshi Yamamoto, Tsuyoshi Takahashi, Yukinori Kurokawa, Kiyokazu Nakajima, Eiichi Morii, Hidetoshi Eguchi, Yuichiro Doki

**Affiliations:** 1 Department of Gastroenterological Surgery, Graduate School of Medicine, Osaka University, Suita City, Osaka, Japan; 2 Department of Gastroenterological Surgery, Toyonaka Municipal Hospital, Toyonaka City, Osaka, Japan; 3 Department of Pathology, Graduate School of Medicine, Osaka University, Suita City, Osaka, Japan; 4 Department of Molecular Pathology, Hyogo Medical University, Nishinomiya City, Hyogo, Japan

## Abstract

**Background:**

The association between tumor-infiltrating lymphocytes and tumor immunity has long been recognized. Among T-cell types, CD45RO-positive memory T cells (CD45RO^+^) are reported to correlate with survival in several cancer types, but clinical evidence is lacking in esophageal squamous cell carcinoma (ESCC).

**Methods:**

In surgical specimens from 162 preoperatively untreated patients, immunohistochemistry for CD45RO was performed to evaluate the density of CD45RO^+^ in the tumor core (CT) and invasive margin (IM) using an auto-count method. Patients were classified into high- versus low-CD45RO^+^ groups based on CD45RO^+^ density in CT and IM separately and combined. The relationship between CD45RO^+^ density and clinicopathological factors, including prognosis, was evaluated.

**Results:**

Average CD45RO^+^ density was 133/mm^2^ in CT and 372/mm^2^ in IM. No significant differences in clinicopathological factors according to high- versus low-CD45RO^+^ scores were identified. Using CT scores, the CD45RO^+^-high group had a better 5-year overall survival (OS) rate (77.2% vs. 54.7% CD45RO^+^-low, *P* = 0.0433), but OS rates did not differ statistically between the two groups by IM scores (75.7% vs. 50.3%, *P* = 0.0576). Using immunohistochemical scores for CT+IM, the survival difference was significant, with a 5-year OS rate of 73.7% for the CD45RO^+^-high group versus 46.3% for the CD45RO^+^-low group (*P* = 0.0141). Multivariate analysis identified CD45RO^+^ CT+IM density as an independent prognostic variable in OS (hazard ratio 2.27, 95% confidence interval 1.43-3.62, *P* = 0.0006).

**Conclusions:**

Density of CD45RO^+^ expression in the CT and IM might be a predictor of long-term survival in ESCC.

**Supplementary Information:**

The online version contains supplementary material available at 10.1245/s10434-024-16530-z.

Esophageal cancer (EC) is the eighth most common malignancy in the world; esophageal squamous cell carcinoma (ESCC) accounts for most cases in Asian countries.^1,2^ Currently, multimodal treatment approaches, including surgery, radiotherapy, and chemotherapy, have been developed, especially for advanced ESCC. In particular, treatment with immune checkpoint inhibitors (ICI) has attracted attention.^3–13^

The importance of the tumor immune microenvironment has been reemphasized in recent years as evidence has grown regarding the therapeutic effect of ICI for several cancer types. The tumor immune microenvironment consists of many host cells, including cytotoxic or regulatory T cells, dendritic cells, macrophages (M1 and M2), B cells, and myeloid-derived suppressor cells.^14^ Among these types, tumor-infiltrating lymphocytes (TILs) have long been reported to play a central role in antitumor immunity.^15–17^ Tumor-infiltrating lymphocytes consist of effector T cells, including CD4+ helper and CD8+ cytotoxic T cells that secrete cytokines and memory T cells that express an isoform called CD45RO.^18^

Human CD45 is a transmembrane tyrosine phosphatase present on all cells of hematopoietic origin except red blood cells and is expressed as CD45RA on most naive T cells. Memory T cells differentiated from their naive T cells express another isoform called CD45RO. Memory T cells can survive for long periods in a quiescent state and have a role in the rapid protective immune response to antigens. Densities of memory T cells that are positive for CD45RO correlate with prognosis in several cancer types,^19,20^ but few reports describe the association in ESCC. Therefore, this study was designed to evaluate the clinical significance of CD45RO positivity in surgical specimens from preoperatively untreated patients with ESCC.

## Material and Methods

### Patients

A total of 162 consecutive patients with preoperatively untreated ESCC who underwent curative esophagectomy at Osaka University Hospital during March 2000 to September 2017 were enrolled in the study (Table 1). All formalin-fixed paraffin-embedded (FFPE) tissues containing the deepest part of the tumor obtained by resection were used for immunohistochemical (IHC) staining and analysis.^21,22^ Cases with other EC types or multiple cancers were excluded. Many of the patients in this study were intolerant to neoadjuvant chemotherapy because of advanced age or renal dysfunction, and ICI as adjuvant therapy was not approved by insurance in Japan. For these reasons, only two patients actually received adjuvant chemotherapy with the fluorouracil-plus-cisplatin regimen. Clinical and pathological data were obtained through medical charts and pathology reports. Information on patient outcomes and survival data also was collected. Tumor stage was classified according to the 8th edition of the UICC/AJCC (Union for International Cancer Control/American Joint Committee on Cancer) TNM classification system.^23,24^ This study was performed with the approval of the Ethics Committee of Osaka University Hospital, and informed consent was obtained from all participants.

**Table 1:** Patient characteristics (N = 162)

		N (%)*
Age	Median (range)	68 (49–85)
Sex	Male	134 (82.7)
	Female	28 (17.3)
Tumor location	Ut	25 (15.4)
	Mt	91 (56.2)
	Lt	25 (28.4)
Histological differentiation (squamous cell carcinoma)	Well	36 (22.2)
	Moderately	102 (63.0)
	Poor	19 (11.7)
	Other	5 (3.1)
pT	1	83 (51.2)
	2	20 (12.3)
	3	56 (34.6)
	4	3 (1.9)
pN	0	83 (51.2)
	1	44 (27.2)
	2	23 (14.2)
	3	12 (7.4)
pM	0	155 (95.7)
	1	7 (4.3)
pStage	I	69 (42.6)
	II	38 (23.5)
	III	48 (29.6)
	IV	7 (4.3)
Lymphatic invasion	0	54 (33.3)
	1	63 (38.9)
	2	42 (25.9)
	3	3 (1.9)
Vascular invasion	0	97 (60.0)
	1	53 (32.7)
	2	8 (4.9)
	3	4 (2.4)

*Ut* upper thoracic esophagus; *Mt* middle thoracic esophagus; *Lt* lower thoracic esophagus;

*Unless otherwise noted

### IHC of CD45RO

A pathologist (K.O.) unaware of the clinical data selected all of the FFPE tissues containing the deepest part of the tumor and invasive margin. The distribution and density of CD45RO-positive memory T cells (CD45RO^+^) in the specimens were evaluated by using IHC with affinity-purified mouse monoclonal antibodies against CD45RO (anti-CD45RO antibody [UCH-L1], Abcam, 1:1000 dilution). The specificity of these monoclonal antibodies in IHC on paraffin-embedded samples was confirmed with human tonsil tissue sections as a positive control.

All FFPE tissues were cut into 4-μm sections, deparaffinized in xylene, and rehydrated through an ethanol gradient. For antigen retrieval, the sections were boiled for 20 min in a pressure cooker at 110 °C in antigen-retrieval buffer (pH 6.0). Slides were peroxidase-blocked in 0.3% H_2_O_2_ in methanol for 20 min and then blocked with normal horse serum (S-2000, Vector Laboratories) at room temperature for 20 min in humid boxes, followed by incubation at 4 °C overnight with mouse monoclonal anti-CD45RO. Next, slides were washed with 1% phosphate-buffered saline (PBS) and incubated with secondary antibody (S-2000 and BA-2000, Vector) at room temperature for 20 min. The slides were then washed again with PBS. For staining for biotinylated secondary antibodies, Avidin-Biotin Complex Staining kits were used (Vectastain ABC Kit, PK6100, Vector) at room temperature for 20 min. Slides were again washed with PBS, and the staining was visualized by incubation with DAB (Wako) for approximately 2.0 min. Sections were counterstained with hematoxylin, dehydrated in ethanol, cleared in xylene, and coverslipped.

### Evaluation of CD45RO^+^ Expression in Resected Specimens

Despite the lack of a consistent recommendation for evaluating IHC of CD45RO^+^, several reports describe the method of selecting five tiles in which lymphocytes are clustered in the tissue.^25,26^ An international working group on immuno-oncology biomarkers has suggested different methods for evaluating TILs in several cancer types. Among these is the Immunoscore method, which combines quantification of the number of TILs in the core of the tumor (CT) with the counts for the invasive margin (IM). This system has recently attracted attention, especially in colorectal cancer, and was applied here.^27–30^

**Fig. 1:**
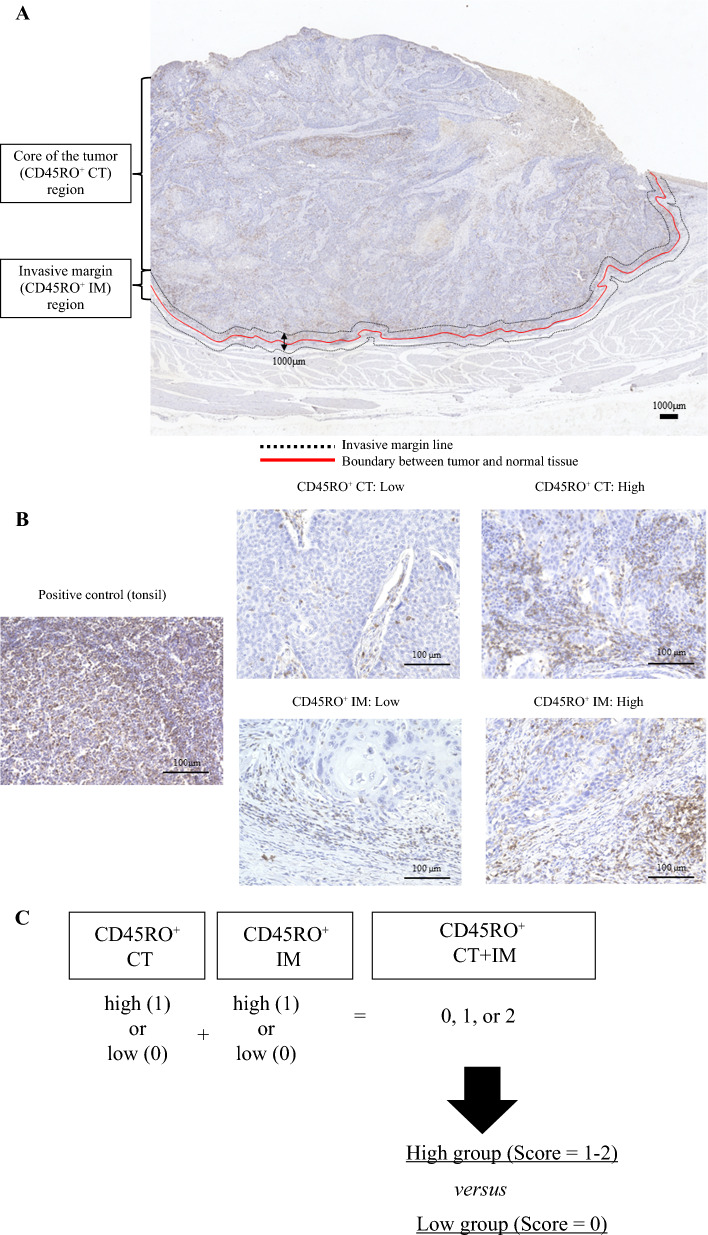
**A** Representative CD45RO^+^ immunostaining section from a resected EC specimen indicating typical tumor regions: core of tumor (CT) and invasive margin (IM). **B** Positive control (tonsil) and representative slides of the low- and high-density of CD45RO^+^ lymphocytes in the CT and IM. **C** Schematic of the CD45RO^+^ score model, evaluated based on the Immunoscore concept

CD45RO^+^ was evaluated separately in the CT and IM and in the two regions combined. The CD45RO^+^ IM region was defined as an area 500 μm inward and outward from the boundary between normal tissue and tumor tissue, and the CD45RO^+^ CT region was defined as all tumor areas interior to the CD45RO^+^ IM region (Fig. 1A).^31,32^ Using automated hybrid cell-counting software (BZ-H3C; Keyence), we counted the number of CD45RO^+^ in multiple tiles (from one tiled view of 500 × 500 μm) containing large numbers of stained CD45RO^+^ in the CD45RO^+^ CT and IM of each primary tumor surgical specimen. The five tiles with the greatest numbers of CD45RO^+^ (designated as hotspots) were each selected in each of the two regions (Fig. 1B) and divided into high (a score of 1) and low (a score of 0) group by using the cutoff value of the mean CD45RO^+^ counts for five hotspots based on previous reports.^27,33^ Then, the number of CD45RO^+^ (CT+IM) was scored (0–2 points) by adding each score of CD45RO^+^ CT and CD45RO^+^ IM regions. Finally, the scores were used to classify the patients into two groups—CD45RO^+^-low (score, 0 points) versus CD45RO^+^-high (score, 1–2 points)—and the association of high versus low scores and clinicopathological variables, including survival, was evaluated (Fig. 1C). All slides were assessed independently by two observers (T.N. and T.M.) blinded to the clinicopathological data and then by conference in case of disagreement. One pathologist (K.O.) confirmed the final diagnosis.

### Statistical Analysis

The survival-time distribution was evaluated using the Kaplan–Meier method. To evaluate independent prognostic significance and relative risk, we performed univariate analysis of clinicopathological factors. Any variables that were significant in the univariate analyses were included in multivariate analyses. Cox logistic regression was used to perform the multivariate analyses. We considered a *P* < 0.05 to be statistically significant. All statistical calculations were performed using JMP version 14 software (SAS Institute, Cary, NC).

## Results

### CD45RO Expression in Surgical Specimens of EC

**Fig. 2:**
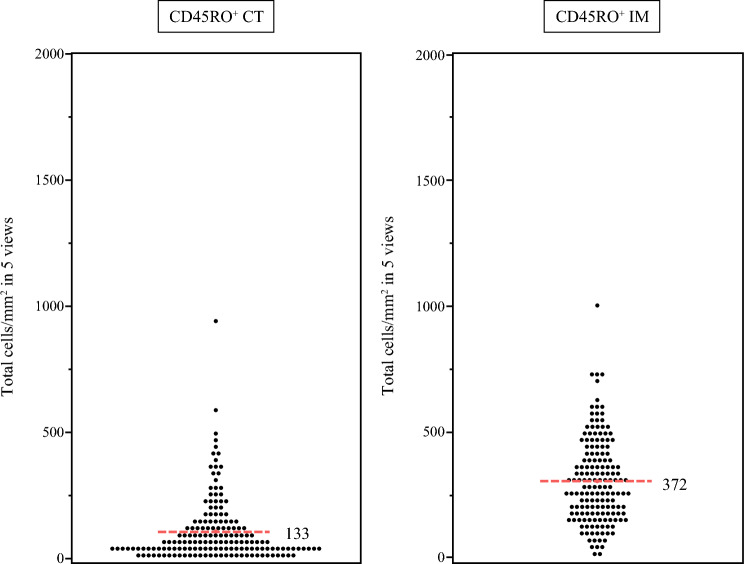
Scatter dot plots of total cell densities (cells/mm^2^) of the top five views used for counting CD45RO^+^ lymphocytes in the CT and IM. Red dotted lines represent the respective average values

A total of 162 samples (Table 1) that contained both CT and IM lesions were evaluated for CD3, CD8, and CD45RO expression by IHC analysis. Supplemental Fig. 1 showed representative slides of CD3^+^, CD8^+^, and CD45RO^+^ lymphocytes in the CT or IM, respectively. The numbers of TILs per area (total cells/mm^2^ in five hotspots) in CD45RO^+^ CT and CD45RO^+^ IM regions on IHC are shown in Fig. 2. Average CD45RO^+^ densities were 133/mm^2^ for CT and 372/mm^2^ for IM.

### Comparison Between CT+IM CD45RO^+^-high and -low Groups and Clinicopathological Parameters

The CD45RO^+^-high group tended to have a lower percentage of lymphatic invasion than the CD45RO^+^-low group (27.5% vs. 40.8%, *P* = 0.0737). However, the two CD45RO^+^ groups did not differ significantly in clinico-pathological variables, including age, sex, tumor location, histological differentiation, pT, pN, pM, pStage, or vascular invasion (Table 2).

**Table 2:** Comparison between CD45RO^+^-high and CD45RO^+^-low groups (combined CT+IM scores) by clinicopathological variables (N = 162)

		CD45RO^+^-high, n = 91; n (%)^*^	CD45RO^+^-low, n = 71; n (%)	*P*
Age	Median (range)	67 (49–85)	69 (49–85)	.4188
Sex	Male	76 (83.5)	58 (81.7)	.7607
	Female	15 (16.5)	13 (18.3)	
Tumor location	Ut	14 (15.4)	11 (15.5)	.9849
	Mt/Lt	77 (84.6)	60 (84.5)	
Histological differentiation (squamous cell carcinoma)	Well/moderately	75 (82.4)	62 (87.3)	.3876
	Poor/other	16 (17.6)	9 (12.7)	
pT	1/2	57 (62.6)	46 (64.8)	.7776
	3/4	34 (37.4)	25 (35.2)	
pN	0/1	71 (78.0)	56 (78.9)	.8960
	2/3	20 (22.0)	15 (21.1)	
pM	0	88 (96.7)	67 (94.4)	.4701
	1	3 (3.3)	4 (5.6)	
pStage	I/II	59 (64.8)	48 (67.6)	.7115
	III/IV	32 (35.2)	23 (32.4)	
Lymphatic invasion	0/1	66 (72.5)	42 (59.2)	.0737
	2/3	25 (27.5)	29 (40.8)	
Vascular invasion	0/1	52 (57.1)	45 (63.4)	.4209
	2/3	39 (42.9)	26 (36.6)	

*Ut* upper thoracic esophagus; *Mt* middle thoracic esophagus; *Lt* lower thoracic esophagus

*Unless otherwise noted

### CD45RO Expression and Overall Survival

**Fig. 3:**
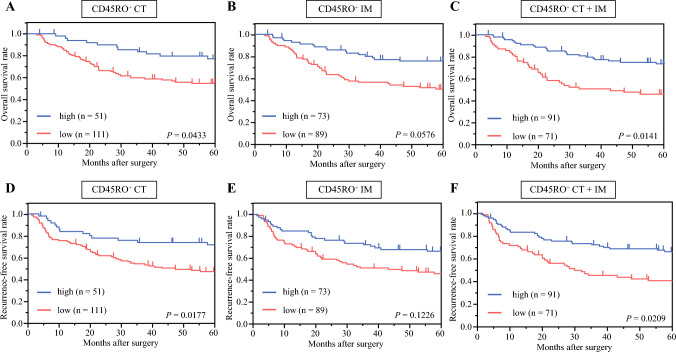
Kaplan–Meier survival curves for overall survival (OS) by high versus low **A** CD45RO^+^ CT scores, **B** CD45RO^+^ IM scores, and **C** CD45RO^+^ CT+IM scores; and recurrence-free survival (RFS) by high versus low **D** CD45RO^+^ CT scores, **E** CD45RO^+^ IM scores, and **F** CD45RO^+^ CT+IM scores

In terms of overall survival (OS), using CT scores, the CD45RO^+^-high group had better 5-year OS rates (77.2% vs. 54.7% for the CD45RO^+^-low group; hazard ratio [HR] = 1.68, *P* = 0.0433), but there was no difference when using IM scores (75.7% high vs. 50.3% low, HR =1.55, *P* = 0.0576). For CT and IM scores combined, the 5-year OS rate was greater for the CD45RO^+^-high compared with the CD45RO^+^-low group (73.7% vs. 46.3%, HR = 1.74, *P* = 0.0141; Fig. 3).

Recurrence-free survival (RFS) results followed a similar pattern. Using CT scores, the CD45RO^+^-high group had a better 5-year RFS rate (71.7% vs. 47.3% for CD45RO^+^-low, HR = 1.80, *P* = 0.0177), but the two groups did not differ in 5-year RFS rates by IM scores (66.2% high vs. 45.8% low, HR = 1.40, *P* = 0.1226). As was the case for OS rates, the 5-year RFS rate was greater for the CD45RO^+^-high group when both CT and IM scores were considered (66.2% vs. 40.5% CD45RO^+^-low, HR = 1.63, *P* = 0.0209).

On univariate analysis, age, pT, pN, pM, lymphatic invasion, vascular invasion, and CD45RO^+^ scores (CT+IM) were statistically significant prognostic factors for OS (Table 3). Multivariate analysis further identified pT (HR = 2.18, 95% confidence interval [CI] 1.22–3.89, *P* = 0.0082), pM (HR = 3.35, 95% CI 1.33–8.43, *P* = 0.0102), and CD45RO^+^ scores for CT and IM combined (HR = 2.27, 95% CI 1.43–3.62, *P* = 0.0006) as independent prognostic factors in OS (Table 3). We have performed multivariate analysis of the RFS data in the same patients and identified CD45RO^+^-CT+IM (HR = 1.95, 95% CI 1.26–3.03, *P* = 0.0028) as an independent prognostic factor along with pT (HR = 2.25, 95% CI 1.29–3.39, *P* = 0.0041), pN (HR = 1.67, 95% CI 1.01–2.76, *P* = 0.0437), and pN (HR = 1.67, 95% CI 1.01–2.76, *P* = 0.0437; Supplemental Table 2).

**Table 3:** Univariate and multivariate analyses of OS (N = 162)

		Univariate		Multivariate	
		HR (95% CI)	*P*	HR (95% CI)	*P*
Age (years)	≥70	2.16 (1.37–3.40)	**.0008**	1.49 (0.92–2.42)	.1083
	<70	1		1	
Sex	Male	1.34 (0.77–2.37)	.3020		
	Female	1			
Location	Ut	1	.4154		
Histological differentiation (squamous cell carcinoma)	Mt/Lt	1.27 (0.71–2.27)			
	Well/moderately	1 1.67 (0.95–2.94)	.0745		
	Poor/other				
pT	1, 2	1	**.0001**	1	**.0082**
	3, 4	3.01 (1.92–4.72)		2.18 (1.22–3.89)	
pN	0, 1	1	**.0001**	1	.1519
	2, 3	2.74 (1.71–4.42)		1.47 (0.87–2.48)	
pM	0	1	**.0002**	1	**.0102**
	1	5.00 (2.15–11.6)		3.35 (1.33–8.43)	
Lymphatic invasion	0	1	**.0009**	1	.0523
	1, 2, 3	2.6 (1.48–4.58)		2.00 (0.99–4.04)	
Vascular invasion	0	1	**.0043**	1	.4316
	1, 2, 3	1.92 (1.22–3.00)		1.25 (0.72–2.18)	
CD45RO^+^ CT+IM score	Low: 0	1.74 (1.11–2.73)	**.0153**	2.27 (1.43–3.62)	**.0006**
	High: 1, 2	1		1	

Bold values indicate statistical significance (*P* < 0.05)

### Patterns of Disease Recurrence

In all cases, the pattern of disease recurrence, including local, lymph node, hematogenous, and disseminated recurrence, was evaluated according to CD45RO^+^ scores for CT and IM combined. Dissemination was observed significantly less in the CD45RO^+^-high compared with the CD45RO^+^-low group (0% vs. 5.1%, *P* = 0.0235; Supplementary Table 1).

## Discussion

In the present study, we evaluated the presence of CD45RO^+^ (i.e., memory T cells) in the CT, IM, and CT+IM of ESCC resection specimens. The density of CD45RO^+^ cells was objectively assessed by using automated cell counting with a digital microscope and hybrid cell counting software. Using scores for the two regions combined, we found that the CD45RO^+^-high group had a better prognosis compared with the CD45RO^+^-low group. CD45RO^+^ in the CT and IM combined was identified in the multivariate analysis as an independent prognostic factor in OS. In addition, this factor was associated with less disseminated recurrence.

In general, memory T cells play an important role in host immune defense against infectious diseases.^34^ Recently, they have been described in association with better prognosis in solid tumors, including gastrointestinal, lung, and breast cancers.^35–38^ Although most of these reports have described high tumor infiltration by CD45RO^+^ as associated with a better prognosis, the mechanism remains unclear. Naive CD8(+) and CD4(+) T cells, after receiving antigen presentation from tumors, differentiate into effector memory T cells, resident memory T cells, and central memory T cells, respectively.^39,40^ CD8(+) central memory T cells are thought to exert antitumor effects mainly by being present in secondary lymphoid organs and releasing cytokines, such as interferon-γ. We evaluated only effector memory T cells or possibly some resident memory T cells in local tissue. Evaluating each subtype of memory T cell separately, along with their function as represented by interferon-γ production, may help elucidate the mechanism.^41^ Also, the causal relationship between CD45RO^+^ expression and disease recurrence was unknown. This study evaluated CD45RO^+^ expression in the primary tumor only. It may be necessary to evaluate CD45RO^+^ expression in the systemic (blood) and lymph nodes to clarify the mechanism of disease recurrence. A previous report on gastric cancer described a correlation between tumor-infiltrating CD45RO^+^ in the primary tumor and peritoneal recurrence (but not lymph node recurrence), which is consistent with our data.^42^

The density of CD45RO^+^ in CT and IM in the present study was evaluated by using the Immunoscore method. Although approaches to evaluating TILs by IHC have varied among cancer types and reports, Immunoscore has been established in the field of colorectal cancer based on reports that TILs within tumors and in the IM, including stromal regions, significantly affect prognosis.^43^ Immunoscore also is useful in several other solid tumors.^15^ Studies using Immunoscore have shown that the CD3/CD8-positive T-cell density ratio of CT to IM varies by cancer type (Supplementary Fig. 1).^44–46^ Few reports have compared CD45RO^+^ cell density between CT and IM, however. We found that the average density of CD45RO^+^ was higher in IM compared with CT. These results may be due in part to differences in tumor stromal volume among cancer types, and although the present study evaluated CT and IM separately, it did not evaluate between stromal and tumor areas. In particular, squamous cell carcinoma involves a tissue structure with a relatively small stromal volume for the tumor nest that may prevent lymphocytes from infiltrating into the tumor.^47^ Our hypothesis was that compared with CT, IM usually plays a more important role in antitumor activity, but we identified the opposite trend in the present study. In our previous work, we found a similar trend in the analysis of CD3- and CD8-positive lymphocytes in esophageal cancer specimens from the same patient cohort, especially in advanced cases.^15^ Conversely, CD3- and CD8-positive T-cell (CD3^+^ and CD8^+^) expression was analyzed in the same patients, and the higher-density group had a better prognosis, especially in advanced cases (pStages II–IV), although not significantly so (5-year OS, CD3^+^ high vs. low: 59.8 vs. 37.5%, *P* = 0.0631; 5-year OS, CD8^+^ high vs. low: 59.6 vs. 39.6%, *P* = 0.3160). Accordingly, among the three lymphocyte subsets, CD45RO^+^ expression was identified as correlating best with prognosis.

This study has several limitations. First, it is a retrospective analysis lacking independent sample validation of the current findings. Second, it included only patients without any preoperative treatments, even though the recent standard of care for locally advanced ESCC is neoadjuvant chemotherapy or chemoradiation, based on pivotal trials.^48–51^ We initially attempted to evaluate CD45RO^+^ in ESCC resection specimens after neoadjuvant chemotherapy, but because of strong tissue modification by the treatment, we found it difficult to distinguish between CT and IM on pathological evaluation. Third, our evaluation of IHC was based on one slide per tumor, using the largest and deepest tumor area of the resected specimen, rather than multiple or all slides. Fourth, we did not perform detailed cell surface marker analysis, such as flow cytometry (FACS) in this study. Therefore, the subtype of CD45RO^+^ that actually affected survival remains unclear. We hope to address this in further studies to clarify the mechanism of the potential role of CD45RO^+^ in esophageal squamous cell carcinoma. Finally, tumor-related immune factors, including PD-L1/2 expression, were not evaluated in the present study.^48^ Simultaneous evaluation of effector T cells, such as CD8-positive and CD4-positive T cells, in addition to regulatory T cells or cytokines as suppressors also may be necessary for a better understanding of the tumor immune microenvironment.^52–54^

## Conclusions

The present results demonstrated that a higher CD45RO^+^ density in resected specimens was associated with significantly better prognosis and less dissemination than a lower CD45RO^+^ density. Although the findings need to be validated in an independent cohort, they may contribute to the establishment of personalized medicine and improvement of clinical outcomes for ESCC patients.

## Supplementary Information

Below is the link to the electronic supplementary material.

Supplementary file1 (TIF 955 kb)

Supplementary file2 (DOCX 18 kb)

Supplementary file3 (DOCX 20 kb)
