# Supplementary figures and images for: CD45RO-Positive Memory T-Cell Density in the Tumoral Core and Invasive Margin Predict Long-Term Survival in Esophageal Squamous Cell Carcinoma
Source: Ann Surg Oncol. 2024 Dec 5;32(3):1953–62. doi: 10.1245/s10434-024-16530-z (PMC11811247; doi:10.1245/s10434-024-16530-z)

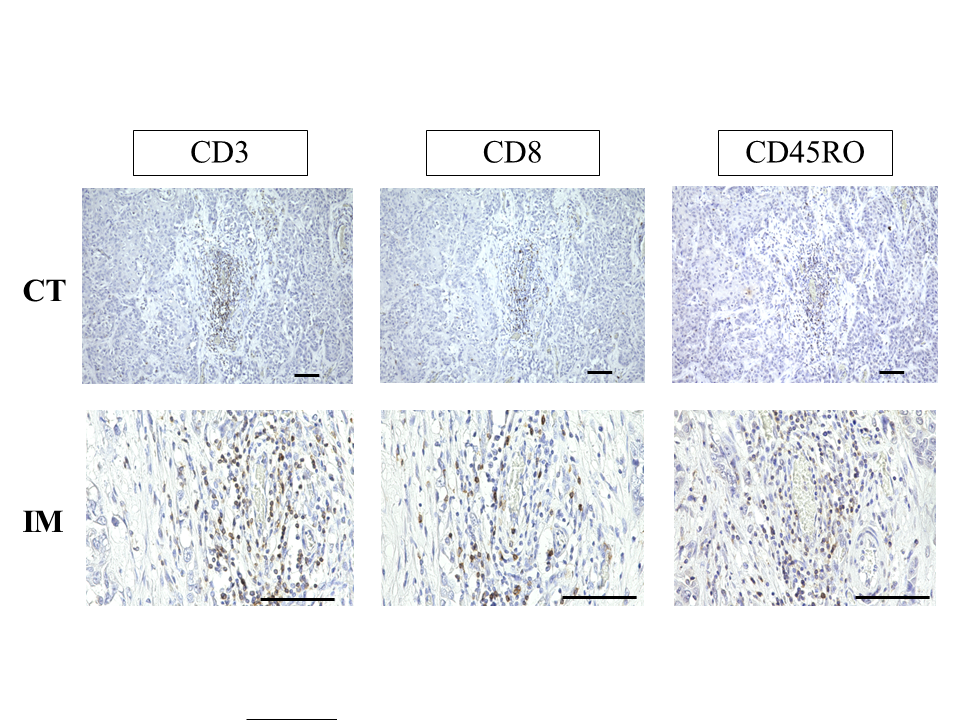

Supplement: Supplementary file 1 — Supplementary file1 (TIF 955 kb) [file 10434_2024_16530_MOESM1_ESM.tif]
