# Supplementary material for: CD45RO-Positive Memory T-Cell Density in the Tumoral Core and Invasive Margin Predict Long-Term Survival in Esophageal Squamous Cell Carcinoma
Source: Ann Surg Oncol. 2024 Dec 5;32(3):1953–62. doi: 10.1245/s10434-024-16530-z (PMC11811247; doi:10.1245/s10434-024-16530-z)
Supplement: Supplementary file 2 — Supplementary file2 (DOCX 18 kb) [file 10434_2024_16530_MOESM2_ESM.docx]

**Supplementary Table 1. CD45RO^+^-high and -low groups (CT+IM scores) and patterns of recurrence (N = 162)**

| Pattern of recurrence | CD45RO^+^ CT+IM  High (n = 64) | CD45RO^+^ CT+IM  Low (n = 98) | *P* |
| --- | --- | --- | --- |
| All recurrence  Present  Absent | 14  15 | 32  66 | .1327 |
| Local recurrence  Present  Absent | 1  63 | 3  95 | .5358 |
| Lymph node recurrence  Present  Absent | 7  57 | 15  83 | .4221 |
| Hematogenous recurrence  Present  Absent | 5  59 | 9  89 | .7601 |
| Disseminated recurrence  Present  Absent | 0  64 | 5  93 | **.0235** |
