# Supplementary material for: CD45RO-Positive Memory T-Cell Density in the Tumoral Core and Invasive Margin Predict Long-Term Survival in Esophageal Squamous Cell Carcinoma
Source: Ann Surg Oncol. 2024 Dec 5;32(3):1953–62. doi: 10.1245/s10434-024-16530-z (PMC11811247; doi:10.1245/s10434-024-16530-z)
Supplement: Supplementary file 3 — Supplementary file3 (DOCX 20 kb) [file 10434_2024_16530_MOESM3_ESM.docx]

**Supplemental TABLE 2. Uni-and multivariate analysis of recurrence free survival in all patients (N = 162)**

|  |  | Univariate analysis | | Multivariate analysis | |
| --- | --- | --- | --- | --- | --- |
|  |  | HR (95% CI) | *P* value | HR (95% CI) | *P* value |
| Age (years) | ≥70  <70 | 2.20 (1.43–3.40)  1 | **0.0003** | 1.50 (0.94–2.39)  1 | 0.0830 |
| Sex | Male  Female | 1.22 (0.71–2.11)  1 | 0.4572 |  |  |
| Location | Ut  Mt / Lt | 1  1.24 (0.71–2.17) | 0.4409 |  |  |
| Histological differentiation (SCC) | well / mod  poor / basoloid | 1  1.58 (0.93–2.70) | 0.0889 |  |  |
| pT | 1, 2  3, 4 | 1  3.20 (2.09–4.90) | **0.0001** | 1  2.25 (1.29–3.93) | **0.0041** |
| pN | 0, 1  2, 3 | 1  2.84 (1.80–4.48) | **0.0001** | 1  1.67 (1.01–2.76) | **0.0437** |
| pM | 0  1 | 1  3.48 (1.50–8.04) | **0.0035** | 1  1.86 (0.75–4.59) | 0.1740 |
| Lymphatic invasion | 0  1, 2, 3 | 1  2.10 (1.27–3.48) | **0.0035** | 1  1.28 (0.66–2.47) | 0.4614 |
| Vascular invasion | 0  1, 2, 3 | 1  2.03 (1.33–3.11) | **0.0010** | 1  1.07 (0.62–1.83) | 0.7973 |
| CD45RO^+^-CT+IM | low: 0  high: 1, 2 | 1.63 (1.07–2.50)  1 | **0.0223** | 1.95 (1.26-3.03)  1 | **0.0028** |
